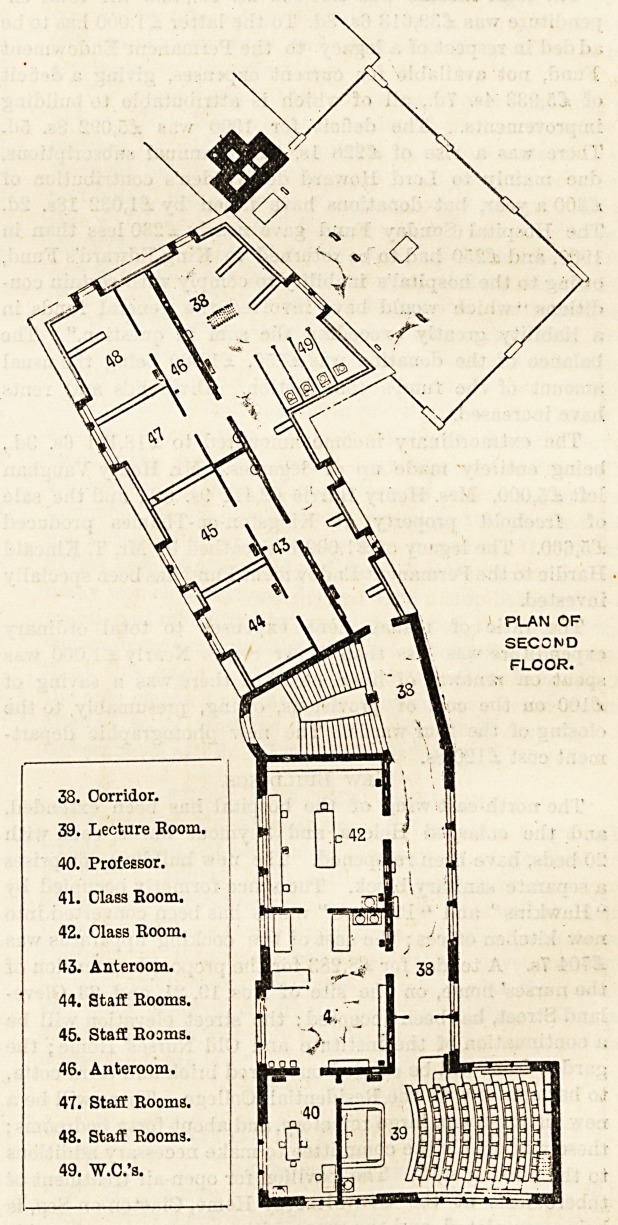# The Royal School for Midwives at Munich

**Published:** 1902-06-28

**Authors:** H. Macnaughton-Jones

**Affiliations:** formerly University Professor in the Queen's University, and Examiner in Midwifery and Diseases of Children in the Royal University of Ireland.


### The Institutional Workshop.

### The Royal School for Midwives at Munich.

By H. Macnaughton-Jones, M.D., M.A.O., F.R.C.S.I. and E., formerly University Professor in the Queen's University, and Examiner in Midwifery and Diseases of Children in the Royal University of Ireland.

I was so impressed by all the arrangements in the School for Midwives at Munich last year, when I was taken over it by Dr. Otto Seitz, that I obtained copies of its rules and regulations, and, after considerable trouble, secured through Frau Dr. Adams Lehmann, from the Minister of Public Affairs, the official plans of the building. From a structural point of view, as indeed from an administrative and educational, I think that this is the most perfect institution of its kind that I have ever been in.

I thought that just at the present time, when the education of midwives and obstetric nurses is attracting so much attention, a brief description of this State institution would be of use in the columns of a journal so specially devoted to the training and interest of nurses generally as The Hospital. I offer here no opinion on the question of midwives' registration. My views on that subject are fairly well known. But I have ever been an advocate for the highest possible education and training of those who undertake the duties either of midwife or obstetric nurse.* And it is mainly from this same standpoint that I consider this short paper may prove of use. Having myself been mainly instrumental in founding a maternity, and having for several years been concerned in the training of midwives, I have always taken a great interest in everything that tends to increase the culture and efficiency of obstetric nurses. This should be the aim and object of every obstetrician. Let us see how it is achieved by the State in Germany.

The Royal School for Midwives, under the directorate of Professor von Winckel and one assistant, is situated immediately behind the Munich Frauenklinik, in the obstetrical department of which some 1,300 births take place per annum. In the school itself there are about 500 to 600 births per annum. It is a building of three stories, with a basement. In the latter are the kitchen, baths, storerooms, with the engine-room and machinery for the lift. On the ground floor, rooms for unsalaried physicians, porters, four large rooms for the students, giving accommodation for forty-five of the latter, and a large tea-kitchen. On ascending to the first floor we reach a large obstetrical ward with cemented walls, in the centre of which is a terraced platform on which the students can stand, and on the same floor are three large rooms, with twenty-one beds for lying-in patients; a bath-room and tea kitchen, with a lecture hall.

On this floor reside the assistant physician, the manager, and the head midwife. On the second floor there is a fine lecture room for the students, and adjoining it are preparation rooms for the lecturer and an examination room, in which examinations are regularly conducted. These rooms, as well as a special working room for the resident physicians, are fitted with various appliances, models, casts, diagrams, etc., in fact, everything necessary for the teaching of midwifery. On the same floor are isolation rooms for any puerperal or other serious cases, besides rooms for servants and attendants. On the third floor we find a room in which are kept all the records connected with the students and the details of all cases treated in the school; also pathological rooms, as well as those for various anatomical preparations and for the different obstetrical appliances required in the school. The entire building is provided with low pressure central heat, with electric ventilation and electric light.

Permission has to be obtained from the Government by every student entering the school, 45 of whom attend each course and live in the building. There are two such courses of systematic instruction in the year, and a third, or recapitulatory, course, each lasting for four months. The teaching is divided

* Vide a "Plea for Obstetric Art," being a paper read before the West London Medico-Chirurgical Society. Med. Press and Cir., Jan. 7, 1891.

into theoretical and practical, and at the end of each of the courses there is an examination, and prizes are awarded to the more distinguished pupils. There are also periodical examinations to test the intelligence and application of the students. Some of the rules are somewhat strict. For example, a penalty is attached for failing to attend a lecture or a delivery, and the strictest silence has to be observed with regard to everything occurring in the establishment, while any quarrelling among the students brings the offenders up for special reprimand. They have to confine themselves to the rooms allotted to them and refrain from loud talking or loitering in any of the corridors. They may not transmit letters, or execute commissions for the patients, nor are they allowed to introduce any article of food for them. Their rooms have to be kept scrupulously clean, and they are responsible for any damage to the furniture. No student may frequent a restaurant or any place of questionable repute. On Sundays and public holidays they have leave of absence from two to five in the winter and two to six in the summer. At all other times they have to obtain permission of the manager before absenting themselves. There are special hours on three days of the week when they may receive visitors. Each student in rotation is liable to a week's dormitory duty, seeing that the dormitories are fit for inspection at any moment, and the student on duty is responsible for the proper ventilation of the rooms. There are also extremely stringent rules with regard to the linen and all articles of clothing worn by the students. They rise at 6 A.M., and take it in turn to superintend at meals, all of which are taken in the dormitories. No light is permitted after 10 o'clock. Three students attend in turn upon every obstetrical case, while one is always in attendance in the obstetrical room or the adjoining preparation room, and sleeps in the latter. When the bell in a dormitory rings, the three students on duty immediately repair to the obstetric room, and if the bell rings a second time the attendance of the whole dormitory is required.

There are strict aseptic and antiseptic precautions enforced. The hair has to be worn closely tied up when on duty, and white linen overalls are worn. Sterilisation of the hands is completed under special instructions, and a student may only undertake an internal examination in the presence of a physician. She has to enter full particulars in a notebook of every case she attends, and be ready to give these particulars verbally at lecture time to the visiting physician.

All through the course of the case she has charge of her patient and the child, under the superintendence of the midwife. Should she infringe any of the rules, the first offence is visited by a reprimand, on the second occasion she receives a warning of dismissal, and should she offend for the third time she is dismissed, though she has the right of

PLAN OF FIRST FLOOR.

14. Lift.
14a. Catering Lift.
27. Accouchement Room.
28. Corridor.
29. Preparation Room.
30. Wards.
31. Wards.
32. Wards.
33. Tea Kitchen.
34. W.C.'s.
35. Surgeon's Assistant.
36. Director.
37. Head Midwife.

appeal to the State. The limits of age for the students are from 20 to 35. They must be fairly well educated, a thorough knowledge of the three R.'s being insisted upon. The main points of instruction are the principles and the carrying out of asepsis and disinfection, the diagnosis of pregnancy, the management of the woman previous to labour, and the care of the child. They are restricted to such operative interference as the management of breech presentations, and they have the practical experience gained by some 200 births during each course. When they have fully satisfied the examiners, they receive the diploma of "Certificated Midwife," the certificate being signed by the director of the school, the assistant, and the Government official. Every two years the midwife has to pass a short test examination conducted by the State physician, and it is clearly understood that she can only practise in what are termed "straightforward" cases.

I have thus sketched the conditions imposed by the State and the limitations enforced on midwives. I have also shown how thorough and complete is the provision made for their education, and how carefully the interests of the public are safeguarded by the limitations imposed.

From my short description but a very imperfect notion can be conceived of the perfection of detail with which this school is conducted, and one cannot but feel regret that, now that the State in England is about to enforce certain conditions on women who adopt the calling of midwife, it is not also prepared, in the three divisions of the Empire, to provide for their technical training and instruction after such manner as is secured for the corresponding class by the State in Germany.

Certainly the creation of such a midwifery school in

PLAN OF SECOND FLOOR.

38. Corridor.
39. Lecture Room.
40. Professor.
41. Class Room.
42. Class Room.
43. Anteroom.
44. Staff Rooms.
45. Staff Rooms.
46. Anteroom.
47. Staff Rooms.
48. Staff Rooms.
49. W.C.'s.

London would tend to raise the standard of the midwife or obstetrical nurse, while her obligation to satisfy State examiners through periodical tests of efficiency would stimulate her to maintain a connection with the school, and to keep in touch with its teaching. This would largely have the effect of increasing the confidence both of the public which employs her, and the medical profession with which she is brought in contact.

## Figures and Tables

**Figure f1:**
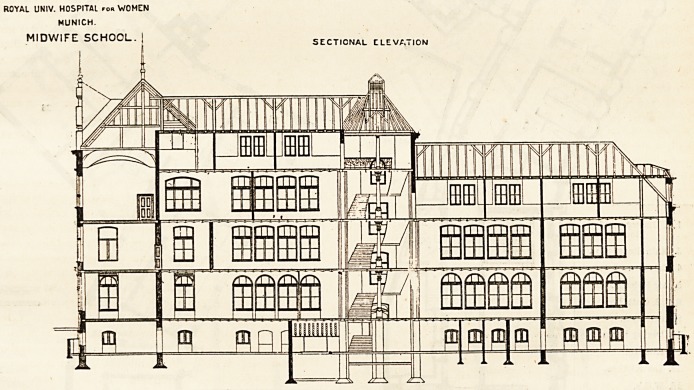


**Figure f2:**
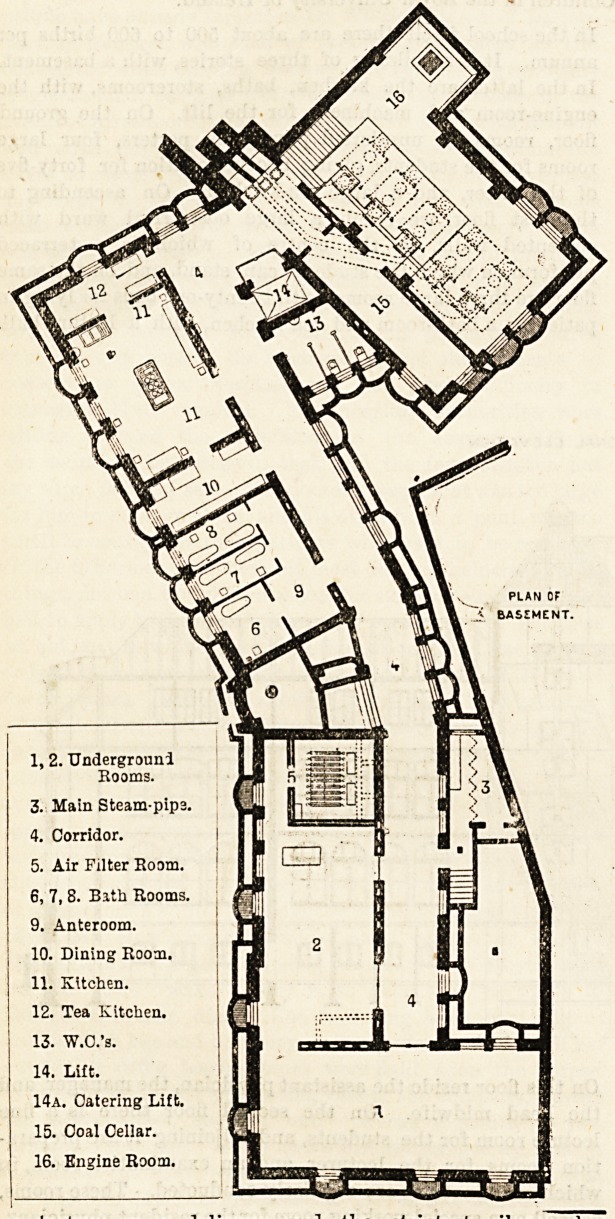


**Figure f3:**
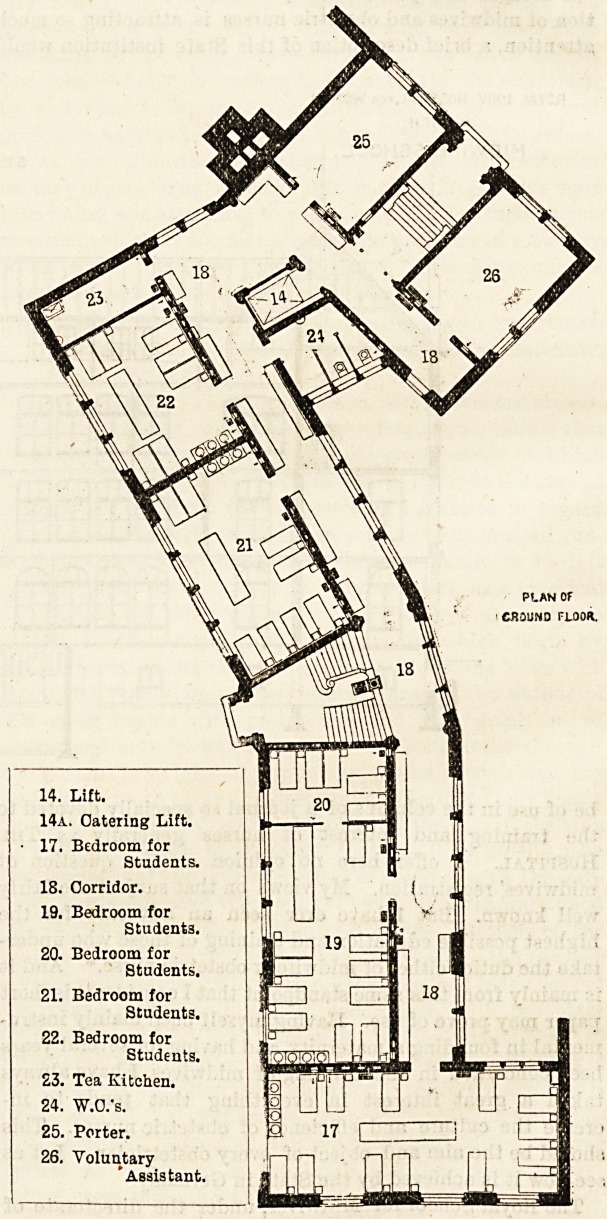


**Figure f4:**
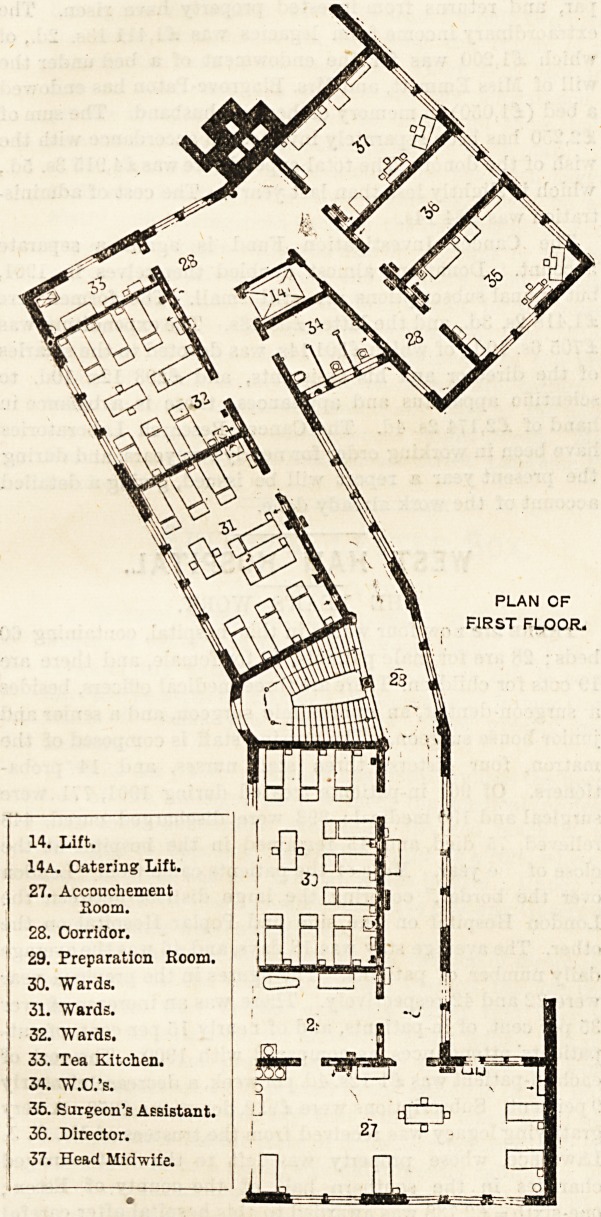


**Figure f5:**